# Visual word identification beyond common words: The role of font and letter case in brand names

**DOI:** 10.3758/s13421-024-01570-3

**Published:** 2024-05-09

**Authors:** Melanie Labusch, Jon Andoni Duñabeitia, Manuel Perea

**Affiliations:** 1 Department of Methodology and ERI-Lectura, Universitat de València, Av. Blasco Ibáñez, 21, 46010 Valencia, Spain; 2 Universidad Nebrija, Madrid, Spain

**Keywords:** Visual word recognition, Instance theories, Abstractionist accounts, Brand names

## Abstract

While abstractionist theories of visual word recognition propose that perceptual elements like font and letter case are filtered out during lexical access, instance-based theories allow for the possibility that these surface details influence this process. To disentangle these accounts, we focused on brand names embedded in logotypes. The consistent visual presentation of brand names may render them much more susceptible to perceptual factors than common words. In the present study, we compared original and modified brand logos, varying in font or letter case. In Experiment 1, participants decided whether the stimuli corresponded to existing brand names or not, regardless of graphical information. In Experiment 2, participants had to categorize existing brand names semantically – whether they corresponded to a brand in the transportation sector or not. Both experiments showed longer response times for the modified brand names, regardless of font or letter-case changes. These findings challenge the notion that only abstract units drive visual word recognition. Instead, they favor those models that assume that, under some circumstances, the traces in lexical memory may contain surface perceptual information.

## Introduction

Visual word identification research, the gateway to reading, has received significant scholarly attention (see Grainger, 2018, 2022, for reviews). Within this domain, the prevailing theoretical position is that the identification of a written word (e.g., *table*) relies on a hierarchical process in which perceptual elements of the visual input (e.g., the visual features of the letters a, A, and *a*) are normalized throughout processing, enabling access to a mental lexicon that is constituted by abstract representations of letter and word units (Coltheart et al., 2001; Davis, 2010; Dehaene et al., 2005; Grainger et al., 2008; Norris, 2006). The underlying idea of these abstractionist accounts in visual word identification is straightforward: When learning how to read, individuals are exposed to letters and words in numerous forms (e.g., different fonts, letter cases, writing styles, etc.). For the sake of efficiency, this variability of the input is normalized throughout the learning process so that the neural representations of letter identities (i.e., the building blocks of words) are stored abstractly (see Grainger et al., 2008; Grainger & Dufau, 2012; Polk et al., 2009). Indeed, there is vast empirical evidence across various paradigms in line with this view. For instance, in masked priming experiments, the target word *EDGE* is processed similarly regardless of whether it is briefly preceded by *edge* or *EDGE*, even though the latter shares both nominal and visual codes (see Jacobs et al., 1995, for behavioral evidence; see Vergara-Martínez et al., 2015, for electrophysiological evidence; see Dehaene et al., 2001, 2004, for neuroimaging evidence). Likewise, the masked mixed-case prime *LaTeRaL* can activate the lexical-semantic representation of a target word (*LATERAL*) equally well as its same-case counterpart *lateral* (Forster, 1998; see also Lee et al., 2002; Perea et al., 2015, for similar evidence). A similar pattern occurs in single-presentation paradigms. In lexical decision experiments, purely visual information about letter identities does not play a role during word processing. For instance, despite the higher visual similarity of the pseudoword *viotin* with its base word (e.g., *t* instead of *l*, in *violin)* than the pseudoword *viocin* (e.g., the letter *l* was replaced with the visually dissimilar letter *c*), the response times and electrophysiological responses to both types of pseudowords are comparable in neurotypical readers (see Perea & Panadero, 2014, for behavioral evidence; see Gutierrez-Sigut et al., 2022, for electrophysiological evidence). If the word processing system had kept some visual information while processing *violin* and *viocin*, one would have expected longer “no” response times, more errors, or a different electrophysiological signature for the visually similar pseudoword *viotin*.

However, the universality of the abstractionist assumption has been called into question by recent studies using logotypes and brand names as printed stimuli (e.g., see Gontijo & Zhang, 2007; Pathak et al., 2019; Perea et al., 2021, 2022). Before reviewing these findings, it is important to note that, unlike common words, brand names (e.g.,  ) are typically written in the same font, color, and letter case configuration (i.e., as logotypes; see Rocabado et al., 2023). All these features are designed to become part of their identity to ease their recognition, and, indeed, even preliterate children can identify popular brand names (Masonheimer et al., 1984). While brand names are constantly present in our modern world (e.g., when going to the supermarket or surfing the web), they differ from common words in both the visual format and the specific contexts in which they occur. The current experiments aim to provide a step toward understanding the identification of brand names and, by extension, their implications for models of visual word recognition.

In a recent experiment, Pathak et al. (2019) found that *anazon* (base word: *amazon*) produced longer responses and more errors than *atazon* using a task in which participants had to decide whether a logotype corresponded to a correctly spelled brand name. Critically, this difference occurs not only with brand names embedded in logotypes but also in brand names presented without format (e.g., in Times New Roman font; see Perea et al., 2022). Furthermore, unformatted brand names are identified faster when their letter case is their prototypical one (e.g., *IKEA* faster than *ikea*, or *adidas* faster than *ADIDAS*; Gontijo et al., 2002; Perea et al., 2015). Similarly, Perea et al. (2022) found an advantage of the intact brand names embedded in logotypes when the brand names were written with a modified font (e.g.,  ). Taken together, these findings favor the view that when identifying brand names, perceptual elements can play a role, thus challenging the universality of abstractionist models of visual word recognition (e.g., Dehaene et al., 2005; Grainger et al., 2008) for which surface visual characteristics such as letters’ font or size details are disregarded early in the word processing stream (e.g., see Chauncey et al., 2008, for the very transient role of font and letter size in masked priming with common words; see also Macaya & Perea, 2014, Slattery & Rayner, 2010, for similar word identification times for across commonly used fonts).

A more general view, not necessarily contradictory to abstractionist accounts, has been provided by the instance theory and its notion of episodic memory traces (see Jamieson et al., 2022, for a recent review), depicting a broader framework to understand the different data patterns with brand names and common words in visual-word recognition experiments. The logic is that each word we encounter is stored as a specific episodic representation that may build upon previous presentations. Hence, when identifying a word, groups of episodic memory traces are activated to access the stored information about previous encounters with that word (Goldinger, 1998; for computational implementations of these ideas, see also Ans et al., 1998; Hintzman, 1986, 1988; Reid et al., 2023). Indeed, in the literature on spoken word recognition, there is evidence that surface features of voice attributes are retained in memory traces for spoken information (e.g., see Clapp et al., 2023; Palmeri et al., 1993). Notably, instance accounts can easily explain why brand names are much more sensitive to perceptual factors than common words: the memory traces of brand names like *amazon* would contain distinct perceptual characteristics with little variability in their perceptual traces (see Rocabado et al., 2023). Instead, common words are encountered in many different formats, leading to a large variability of memory traces (with different fonts, colors, and case configurations). As these different formats in common words would play no linguistic role (e.g., HOUSE, house, or House would refer to the same meaning), their memory representations would not be tied to specific perceptual representations, being *functionally* abstract (see Goldinger, 1998).

In addition, an approach that lies in the middle ground between purely abstractionist and strictly instance-based accounts is proposed by models suggesting the simultaneous operation of multiple generalized processing mechanisms, such as weakly abstractionist accounts and multiple systems accounts. These accounts posit that visual word recognition happens through a dynamic interplay between abstract, higher-level representations and more detailed, perceptual-level representations (e.g., for reviews, see Bowers, 2000; Marsolek, 2004; Tenpenny, 1995). Unlike strong abstractionist accounts that emphasize the dominance of abstract representations, visual word recognition is seen as a flexible and interactive process where both abstract word forms and specific visual features may contribute to comprehension (Bowers, 2000). In this way, weakly abstractionist accounts would acknowledge the importance of perceptual information, such as letter shapes and case configurations in some words, alongside higher-level abstract representations in most other words, thus providing a nuanced understanding of how readers recognize words (Bowers, 2000; Marsolek, 2004; Tenpenny, 1995). In line with this idea, the multiple systems account additionally posits that there may exist various perceptual subsystems, where one system encodes visual shapes in an abstract manner and the other one accounts primarily for perceptual information (see, for discussion, Deason & Marsolek, 2005; Marsolek et al., 1992, 1994, 1996; Marsolek, 2004; Marsolek & Burgund, 2008; Schacter et al., 2004). Such an explanation leaves open the possibility that both, abstract and instance-based components play a part in the internal representations of written words (Bowers, 2000).

The present paper aims to contribute to our understanding of the processing mechanisms underlying written word recognition by looking at a particular class of words with a distinctive surface format: brand names. We focused on two factors assumed to be irrelevant in abstractionist accounts of visual word recognition: font and letter case. Bear in mind that Cohen and Dehaene (2004) explicitly listed these two elements among the “irrelevant” perceptual parameters during lexical access (“position, size, color, font, or case”, p. 466). However, as reviewed earlier, when participants are asked whether a printed item is an existing brand name or not, response times are faster when presented in their usual letter case configuration (e.g., *IKEA* faster than *ikea*; Gontijo et al., 2002; Perea et al., 2015). While these results are, in principle, problematic for abstractionist theories of written word recognition, one might argue that the variant of the lexical decision task employed in these experiments (i.e., does the stimulus correspond to a brand name or not?) could have induced some task-specific post-access verification processes that could benefit the most usual visual format. In lexical decision experiments, same-case words (e.g., HOUSE) are responded to faster than the less visually familiar, mixed-case words (e.g., HouSe), whereas same-case pseudowords (e.g., GUABE) are responded to slower than mixed-case pseudowords (e.g., GuAbE), suggesting a task-specific bias (e.g., “if the letter string appears familiar, it is more likely to be a word,” see Perea et al., 2020). Critically, the difference in response times between words like HOUSE and HouSe vanishes in a semantic categorization task where participants decide whether each presented word referred to an animal name (Perea et al., 2020; see also Laham & Leth-Steensen, 2023). These findings favor the view that the slowdown of HouSe relative to HOUSE in the lexical decision task is mediated by task-specific post-access mechanisms (see Forster, 1998, for converging evidence using masked priming; see also Grainger & Jacobs, 1996, for modeling a familiarity mechanism specific to the lexical decision task).

**Fig. 1:**
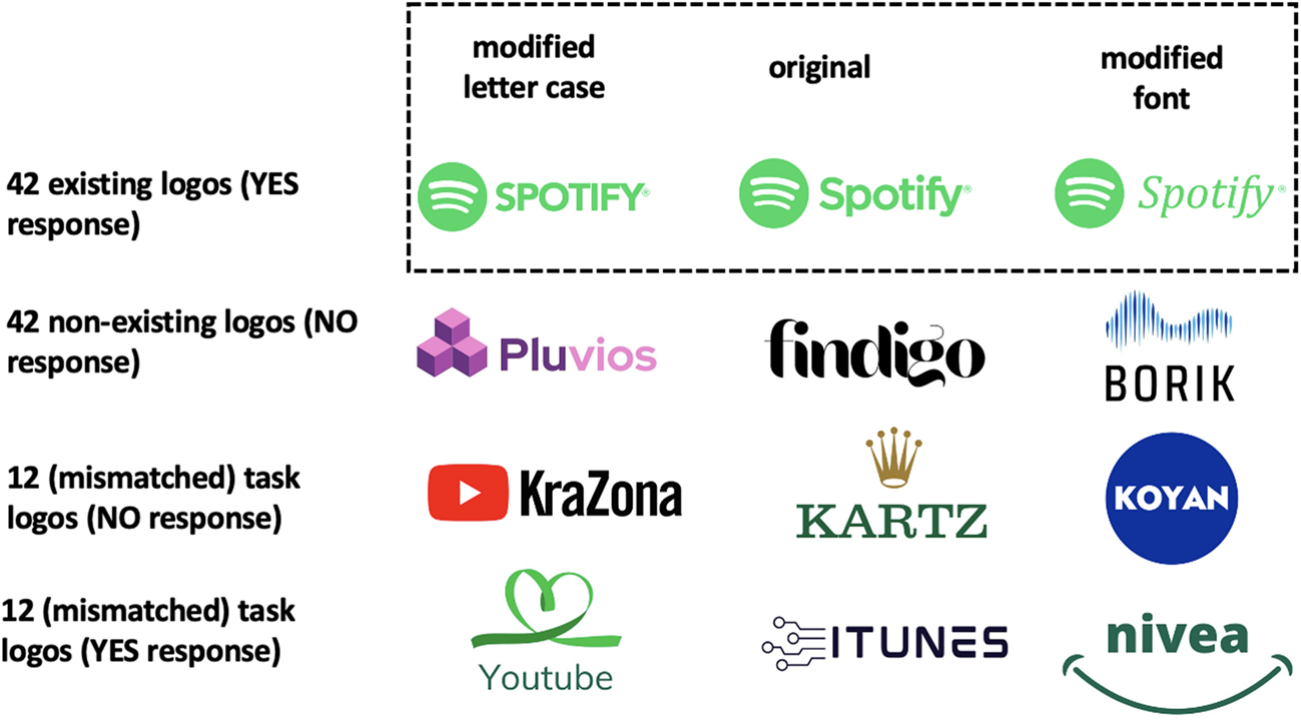
Materials of Experiment 1: We selected 42 known brand names that were presented in three versions: (1) the original version, (2) with a modified letter case, and (3) with a modified font. We additionally created 42 fake brand names and 24 mismatched brand names (real brand names in fake logos and fake brand names in authentic logos) to ensure that participants would pay attention to the written words

Thus, a more conclusive demonstration of the role of letter case and font during the identification of logotypes would be via a task that relies exclusively on a semantic property, not tied to interpretive issues that may occur in lexical decision experiments (see Forster & Shen, 1996, for discussion). To that end, we designed two experiments. In Experiment 1, we asked participants to decide whether the brand name embedded in a logotype existed or not (e.g., the actual brand *amazon* vs. the non-existing brand *pluvios*). This task, which has been used in several previous studies with brand names (see Gontijo et al., 2002; Pathak et al., 2019; Perea et al., 2021, 2022), can be considered a variant of a lexical decision task adapted to the context of brand names. The brand names could be written intact, with a different font or case (see the top panel of Fig. 1). To ensure that participants were processing the written words rather than the graphical content of the logotypes, we included a small proportion of filler items (see the bottom panel of Fig. 1): (1) existing brand names embedded in non-existing logos (i.e., requiring a “yes” response) and (2) non-existing brand names embedded in existing logotypes (i.e., requiring a “no” response). For the experimental trials, we expected to replicate the advantage of the intact format reported in earlier research with unformatted brand names in this task (e.g., Perea et al., 2015, 2022).

In Experiment 2, the critical experiment, we employed a task that relies on semantic information employing the same brand names as in Experiment 1. To provide participants with an easily relatable semantic category that is comparable to the classic “animal” versus “non-animal” or “tools” versus “non-tools” semantic categorization tasks in previous research (e.g., Mayall & Humphreys, 1996), we asked them to decide whether the brand name embedded in a logotype corresponded to a transportation company. Again, the brand names were written intact, with a different font or case. We also included a small proportion of filler items: (1) transportation brand names embedded in non-transportation logos (i.e., requiring a “yes” response) and (2) non-transportation brand names embedded in transportation logos (i.e., requiring a “no” response) (see Fig. 3).

We can deduce the following predictions from the range between abstractionist and instance-based accounts. If font and letter case information of brand names are used as retrieval cues during the identification of logotypes, we expect longer response times to the brand names with modified letter case or font compared to the intact brand names regardless of task (i.e., the same pattern in both experiments). This pattern would challenge purely abstractionist models of visual word recognition (e.g., Dehaene et al., 2005; Grainger et al., 2008), which would predict a null effect. Instead, at least for those stimuli that are often presented in the same format such as brand names, this outcome would favor those models that assume that lexical memory may contain surface characteristics of the stimuli such as font or letter case, as proposed by instance models and weakly abstractionist/multiple systems accounts of word recognition (see Ans et al., 1998; Bowers, 2000; Goldinger, 1998; Kwantes & Mewhort, 1999; Marsolek, 2004; Reichle et al., 2022; Wagenmakers et al., 2004). Alternatively, if the retrieval of perceptual codes such as font and letter case is not a general property during the identification of brand names but is rather task-dependent, we expect the advantage of the intact brand names in the lexical decision task (Experiment 1; i.e., a task that may be more dependent on visual familiarity; see Perea et al., 2020) but not in the semantic categorization experiment (Experiment 2). This latter outcome would constrain the role of surface elements of brand names when accessing lexico-semantic information, thus limiting the importance of perceptual cues during visual word recognition.

## Experiment 1: Lexical decision task

### Methods

#### Participants

We recruited 50 native Spanish individuals (mean age = 28.6 years, SD = 5.6 years, 23 self-identified as women) via Prolific's online recruitment platform (www.prolific.co). This sample size guaranteed 2,100 observations in each condition for the brand names (50 subjects x 42 items/condition). Following Brysbaert and Stevens’ (2018) guidelines, this sample size should be effective in detecting even small effects. All participants reported no reading/writing problems and corrected/normal vision. Participants received monetary compensation according to the average pay rate per hour from Prolific. Before the experiment, each participant gave informed consent to participate in the study. The Research Ethics Committee of the University of València approved the experiments, per the principles stated in the Declaration of Helsinki.

#### Materials

We selected 42 commonly known brand names. None of them was a common word from the English or Spanish dictionary (e.g., the brand name *Puma* was excluded because it is also a common word (i.e., an animal)). To ensure the brands were familiar to all participants, we conducted a pre-study with ten native Spanish individuals who fulfilled the same recruitment criteria as in the experiment (mean age = 26.7 years, SD = 4.6 years, four self-identified as women). In the pre-study, participants were asked to rate 106 commonly known brand names according to their familiarity on a scale from 1 = "completely unfamiliar" to 5 = "completely familiar." As a control, we also included nine unknown brand names. The average familiarity ratings per item gave us a familiarity index for each brand name. We selected the highest-scoring brand names for our experiment. Appendix 1 presents the full results of the pre-study, and Appendix 2 includes the list of the items in the experiment. Each brand name was presented in three versions: (1) the original version; (2) with a modified letter case, i.e., when the logo was written in uppercase letters, in this version, it was written in lowercase letters, using the same font; (3) with a modified font that significantly differed from the original font, maintaining the original letter case configuration (see Fig. 1). Brand names with a single uppercase letter in the beginning or middle of their name were treated as lowercase letter brand names. Hence, their letter-case modification resulted in a full uppercase letter format.^1^

Additionally, we created 42 non-existent brand names with logos. The brand names were created with an artificial language model (OpenAI, 2023), following the criteria of popular brands in different sectors and individually adjusted manually. Note that for generating pseudo-brand names, it is not possible to apply classical pseudoword generation programs (e.g., *Wuggy*; Keuleers & Brysbaert, 2010) as brand names usually do not follow the Spanish orthography (e.g., the name of the Spanish airline *vueling*, which contains the English suffix -*ing*). We cross-checked each artificial brand name in the European Union Intellectual Property Office (EUIPO) database (https://euipo.europa.eu/eSearch/) to ensure that no invented brand names were registered as existing trademark brands (as of April 2023). We used standard logo templates from the online graphic website “Canva” (www.canva.com) for the logo designs. We manually adjusted the aesthetics of the fake logos to make them visually comparable to the actual brand names. We assessed this through a carefully conducted visual inspection of the non-existent brand names in relation to the corresponding set of existing brand names. The non-existent brand names were matched in word length (mean word length = 7.3 letters, minimum length = 4 letters, maximum length = 16 letters) and letter case (34 lowercase letter brands, 20 uppercase letter brands) with the set of genuine brand names.

To ensure that participants read the brand names and did not make their decision by merely looking at the graphic design, we added 24 filler brands where a novel set of brand names did not match their logos. For instance, the cosmetics brand NIVEA was presented with a non-existent logo, and the non-existent brand KOYAN was presented within the NIVEA logo (see Fig. 1). Half of the filler items were existing brand names that had not occurred in the experiment before, within non-existing graphical designs of logos (“yes” response), and the other half were non-existing brand names within existing graphical designs logos (“no” response) that had not occurred in the experiment before.

#### Procedure

The experiment was programmed with PsychoPy 3 (Peirce et al., 2022) and hosted online on Pavlovia (www.pavlovia.org). Participants were asked to be in a quiet room without any distractions during the experiment. Each participant saw one brand name with its logo at a time and was instructed to categorize it as “existing” or “non-existing” by pressing the “M” or “Z” buttons, respectively, on their keyboard. We asked participants to pay specific attention to the written words. If the fake brand “Vezor” was presented within the logo of the actual brand “Gucci,” participants were to respond “non-existing.”

Before the start of the experiment, there were 14 practice trials with feedback to familiarize the participants with the task. A trial consisted of the presentation of a fixation cross for 50 ms and the presentation of the brand name until a response (or until a deadline of 2,000 ms). The experiment was composed of three blocks. Each existing brand name occurred only once in each block and only once in each of its three forms (original, modified letter case, modified font) throughout the experiment. The non-existing and filler brand names were always presented in the same form. The order of presentation of the items within each block and the order of the blocks were randomized. Each block consisted of 108 items: 42 existing brand names, 42 non-existing brand names, and 24 filler stimuli (50% “yes” response, 50% “no” response). This resulted in a total of 324 trials across the three blocks. There were breaks between each block, and the median completion time of the experiment was around 11 min.

#### Data analysis

We analyzed the data from the experimental trials (i.e., those with real brand names and in which their graphical design, other than letter case or font, was kept) with Bayesian linear mixed-effects models in R (R Core Team, 2023) using the *brms* package in Stan (Bürkner, 2017; Stan Development Team, 2023). The only fixed factor of the models was Format (intact, modified letter case, modified font) in which the reference condition was “intact.” This allowed us to examine the potential decisional cost when the letter case was modified (intact vs. modified letter case) or the font was modified (intact vs. modified font). The random factor structure was the maximal (i.e., items’ and participants’ intercepts and slopes for Format). As response time data distributions have a positive skew, we used the ex-Gaussian distribution to model the latency data (Ratcliff, 1993). The accuracy data were modeled with the Bernoulli distribution due to their binary nature (Ratcliff & Rouder, 1998). Each model consisted of four chains, with 5,000 iterations (warmup: 1,000 iterations) in each chain. We used the default priors from brms. For the output, these models indicate the coefficient (*b*) of each effect (i.e., the mean of the posterior distribution), its estimation error (i.e., the standard error of the posterior distribution), and its 95% credible intervals. Evidence of a decisional cost would be reflected in coefficients falling beyond the 95% credible interval.

### Results and discussion

For the latency analysis of the experimental trials, we removed incorrect trials and very short responses (less than 250 ms; four data points, less than 0.01%) from the dataset. Responses of more than 2,000 ms (i.e., the response deadline) were automatically classified as incorrect and removed. The descriptive statistics of the mean response times and mean error rates are given in Table 1. Both the latency and accuracy linear mixed-effects models converged successfully (all R̂s were 1.00).

**Table 1:** Mean response times (in ms) and error rates (in percentages) for existing brands written in their original form, with a modified letter case and a modified font in Experiment 1. Although not relevant to the present analyses, we also included the mean response times and error rates for non-existing brands and the filler trials

	Response time (in ms)	Error rate (in percentage)
Original	577	5.5
Modified letter case	610	5.9
Modified font	610	6.0
Non-existing brands	677	2.6
Filler trials	698	12.0

#### Response times

**Fig. 2:**
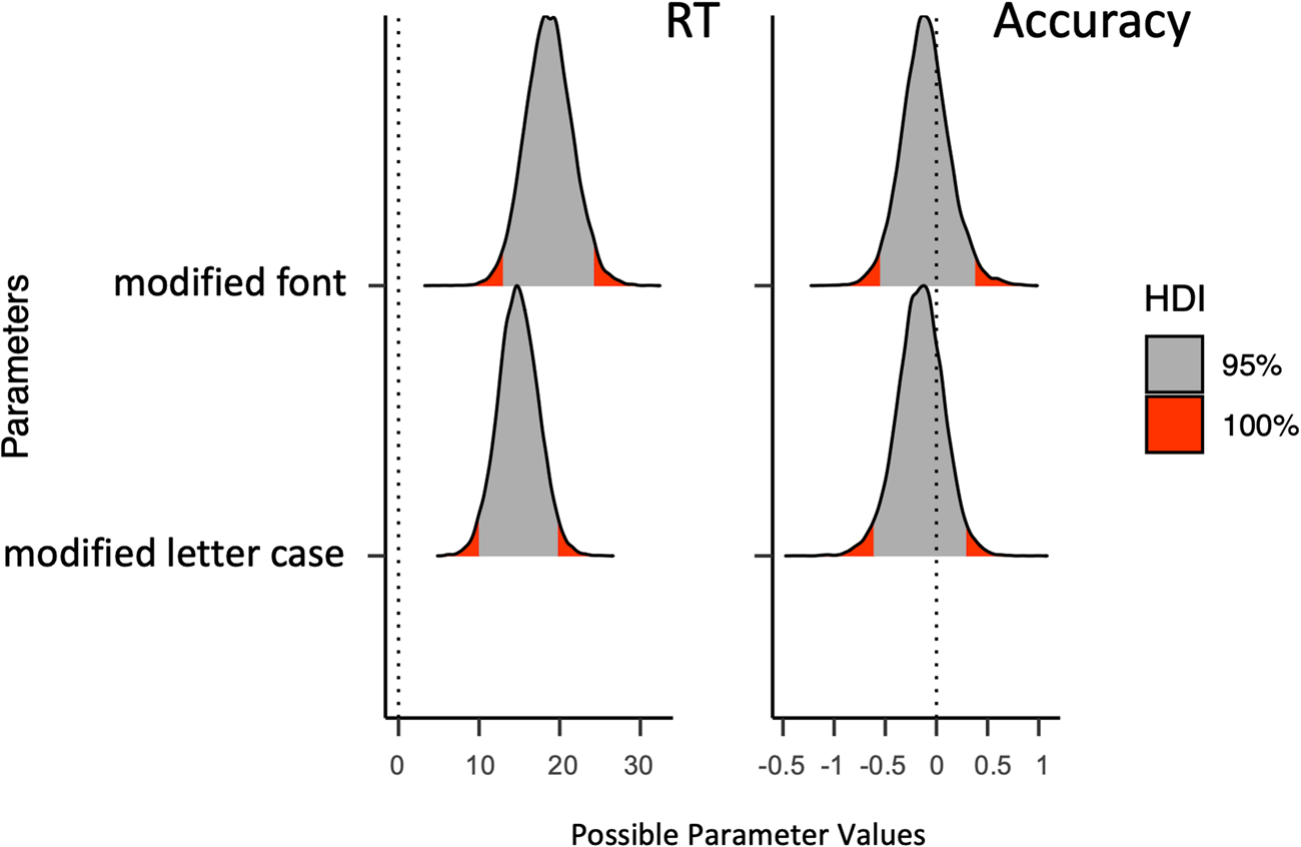
Highest density intervals with the 95% and 100% credible intervals of the posterior distributions for each of the estimates of the Bayesian linear mixed-effects models on response time (**left panel**) and accuracy (**right panel**) for the logotypes in Experiment 1. The reference condition was the original logotype, which was compared to logotypes with a modified font and logotypes with a modified letter case

Relative to the intact logotypes, we found longer response times when the brand names were written in a modified font (*b* = 18.66, *Estim.Error* = 2.90, *95% CrI* [13.07, 24.41]) or a modified letter case (*b* = 14.85, *Estim.Error* = 2.50, *95% CrI* [9.96, 19.79]). For the posterior distributions, see Fig. 2.

#### Accuracy

The intact brand names produced similar accuracy to the brand names with a modified font (*b* = -0.1, *Estim.Error* = 0.23, *95% CrI* [-0.55, 0.38]) or letter case (*b* = -0.16, *Estim.Error* = 0.23, *95% CrI* [-0.63, 0.28]).

The present experiment used a variant of a lexical decision task with brand names, where participants had to decide whether the item corresponded to an existing brand name. We found longer response times for the logotypes where the letter case or the font was modified from the original logotypes (see Fig. 2), replicating earlier research (e.g., modified letter case: Gontijo et al., 2002; Perea et al., 2015; modified font: Perea et al., 2022).^2^

While the observed cost with the modified font or letter case of brand names favors episodic over abstractionist accounts of visual word recognition, one might argue that, in lexical decision tasks, participants could have used some task-specific processes to help to discriminate between existing brand names over the non-existing brand names. For instance, the standard brand names could have greater visual familiarity than the modified brand names, thus speeding up the responses, in the same way than in lexical decision the same-case word *LATERAL* is responded to faster than the mixed-case word *LaTeRaL* (e.g., see Perea et al., 2020). Similarly, some non-existing brand names could be perceived as less readable than the actual ones, especially for those of presumed foreign origin. These potential task-specific issues reinforce the need to examine the effects of font and letter case with brand names in a purely semantic task with existing brands, as was done in Experiment 2.

## Experiment 2: Semantic categorization task

The critical question in Experiment 2 is whether the processing disadvantage of the modified brand names (in terms of font or letter case) that occur in lexical decision experiments – as also shown in Experiment 1 – can be generalized to a task that requires access to lexical-semantic information. Keep in mind that, with common words, the familiarity of the visual format – in the form of the letter case – plays a role in the lexical decision task but not in a task that relies on semantics. For instance, as noted earlier, the disadvantage that occurs for common words in unfamiliar formats (e.g., mixed-case words such as *hOuSe*) over familiar formats (e.g., same-case words such as *HOUSE*) in lexical decision tasks vanishes in semantic categorization tasks (e.g., animal vs. non-animal word; see Perea et al., 2020).

**Fig. 3:**
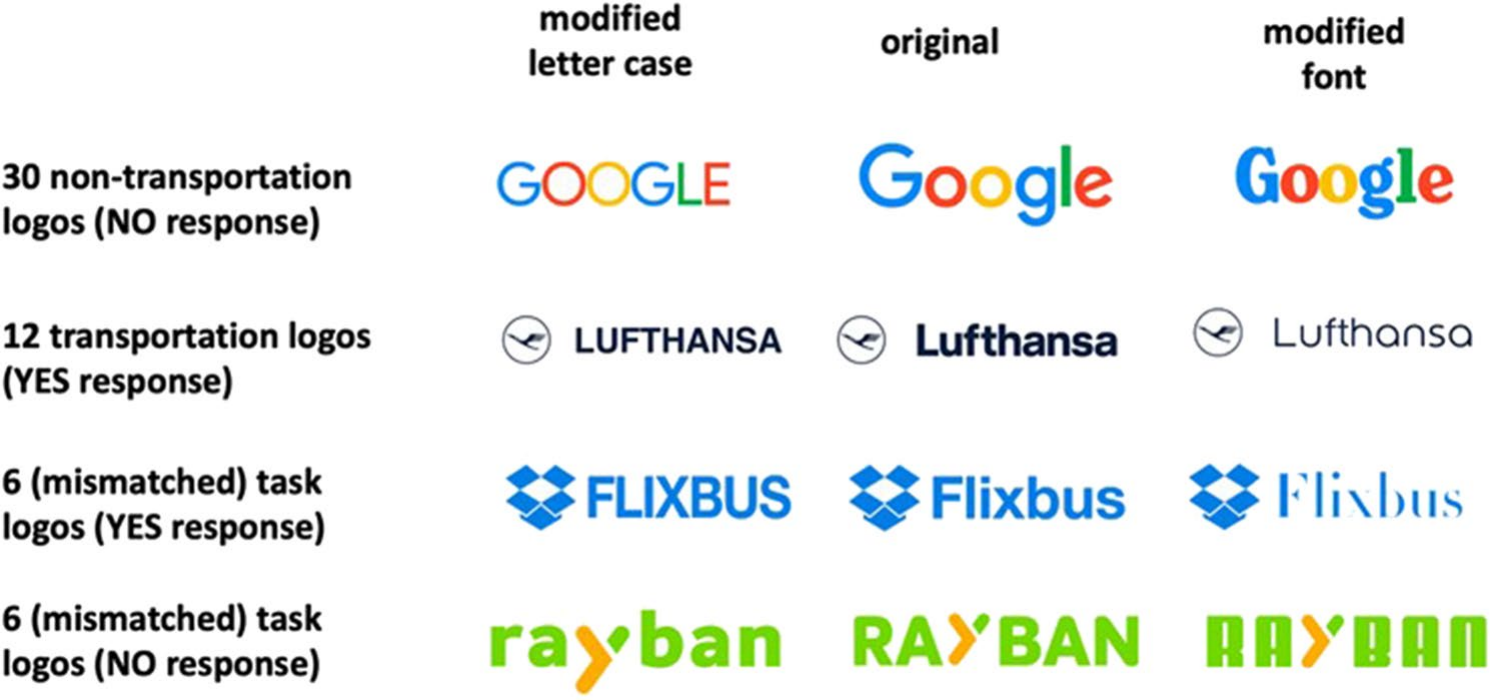
Materials of Experiment 2: We chose the same 42 brand names as in Experiment 1, of which 30 were non-transportation brands (66.6%) and 12 were transportation brands (33.3%). We included 12 filler mismatched brands (transportation brand in non-transportation logo and vice versa) to ensure that participants would pay attention to the written words. The logos were presented in their three versions: (1) modified letter case, (2) modified font, and (3) original. For the mismatched brands, the “original” version kept the graphical design (font and letter case) of the brand name that was supposed to be represented

In Experiment 2, we used the same brand names as in Experiment 1, but the participant's task was to decide whether the brand name referred to a means of transportation (i.e., a semantic categorization task). We chose transportation and non-transportation brand names as semantic categories because participants are typically exposed to brands corresponding to means of transportation in their everyday lives. Thus, whether a brand name corresponds to a means of transportation is widely known, and, therefore, participants should be making this categorization without difficulty. To make sure that participants had to read the brand names to correctly perform the task, as depicted in Fig. 3, a small number of brand names were purposely embedded with a graphical design that corresponded to the other category (e.g., the transportation brand *Flixbus* with the logo of *Dropbox*, or the non-transportation brand *Ray Ban* with the logo of *Flixbus*^3^).

To sum up, the goal of Experiment 2 was to find out whether the visual information of brand names is also processed in a task that relies on lexico-semantic information. To that end, participants had to categorize the brand names embedded in logotypes as belonging or not to a particular semantic category. In the present experiment, the semantic categories consisted of brand names belonging to a transportation company (e.g., *Lufthansa*) or brands that did not belong to a transportation company (e.g., *Google*) (see Fig. 3). The predictions were clear-cut. The presence of slower response times for brand names presented with a modified font or a modified letter case relative to the intact logotypes suggests that the access to brand names in the mental lexicon uses perceptual codes, favoring episodic accounts of visual word recognition. Alternatively, similar response times for the modified and intact logotypes would favor abstractionist accounts. Furthermore, this latter outcome would suggest that prior empirical evidence of perceptual factors in brand names with tasks requiring deciding whether an item is a brand name (e.g., GUCCI vs. VEZOR) – including that of Experiment 1 – could have been task-dependent.

### Methods

#### Participants

We recruited an additional set of 50 native Spanish individuals (mean age = 27 years, SD = 4.8 years, 25 self-identified as women) with the same recruitment criteria as in Experiment 1.

#### Materials

We used the same 42 commonly known brand names as in Experiment 1. Twelve belonged to a means of transportation, whereas 30 were brands that could not be associated with a means of transportation. As in Experiment 1, we added 12 filler brands (six transportation and six non-transportation brands) where the brand names did not match their logos (e.g., the soft drink brand *Fanta* was presented within the logo of the car rental company *Hertz*, see Fig. 3). Again, each brand name was presented in three versions: (1) the original version, (2) with a modified letter case, and (3) with a modified font (see Fig. 3).

#### Procedure

The experimental procedure was the same as in Experiment 1, except that participants were instructed to perform a semantic categorization task. Participants were asked to categorize the brand names as a “means of transportation” or “no means of transportation.” Again, we asked participants to pay specific attention to the brand names embedded in the logos. Hence, if the transportation brand *Uber* was presented within a non-transportation brand logo (e.g., *Nike*), participants should respond, “means of transportation.” Each experimental block consisted of 54 items (33.3% “yes” response, 66.6% “no” response), resulting in a total of 162 trials.

#### Data analysis

The data analyses were the same as in Experiment 1, except that the models included a second fixed factor: Type of Brand (Transportation, Non-transportation). Both, the latency and accuracy models had the maximal random-effect structure in the design: (1+ Format * Type_of_Brand |subject) + (1+ Format |item).

### Results and discussion

We excluded incorrect responses and very short response times (< 250 ms; one data point, less than 0.01%) from the response time analysis. The mean response times and error rates are presented in Table 2. All Bayesian linear mixed-effects models on the latency and accuracy data produced good fits (all R̂s = 1.00).

**Table 2:** Mean response times (in ms) and error rates (in percentages) for non-transportation brands and transportation brands written in their original form, with a modified letter case, and with a modified font in Experiment 2

	Non-transportation brands		Transportation brands	
	Response time	Error rate	Response time	Error rate
Original	622	2.2	684	6.8
Modified letter case	631	2.8	692	5.2
Modified font	635	2.7	726	8.2

#### Response time analysis

We found faster responses for the non-transportation than for the transportation brands (*b* = -62.05, *Estim.Error* = 12.02, *95% CrI* [-85.72, -38.50]). More importantly, compared to the intact logotypes, we found longer response times when the brand names had a modified letter case (*b* = 11.86, *Estim.Error* = 5.05, *95% CrI* [2.03, 21.76]). This effect was similar for transportation and non-transportation brand names (interaction: *b* = 2.0, *Estim.Error* = 5.89, *95% CrI* [-9.46, 13.62]). We also found longer response times for the brand names with a modified font relative to the intact brand names (*b* = 28.83, *Estim.Error* = 6.0, *95% CrI* [17.27, 40.79]). Although this cost due to the modified font was slightly larger for transportation brand names than for non-transportation brand names, we prefer to remain cautious about this interaction: (1) its coefficient was barely outside the 95% Credible Interval (interaction: *b* = -13.51, *Estim.Error* = 6.74, *95% CrI* [-26.86, -0.28]), and (2) part of the effect was due to a 198-ms difference in one of the items (Avianca, a Latin American airline).

#### Accuracy analysis

**Fig. 4:**
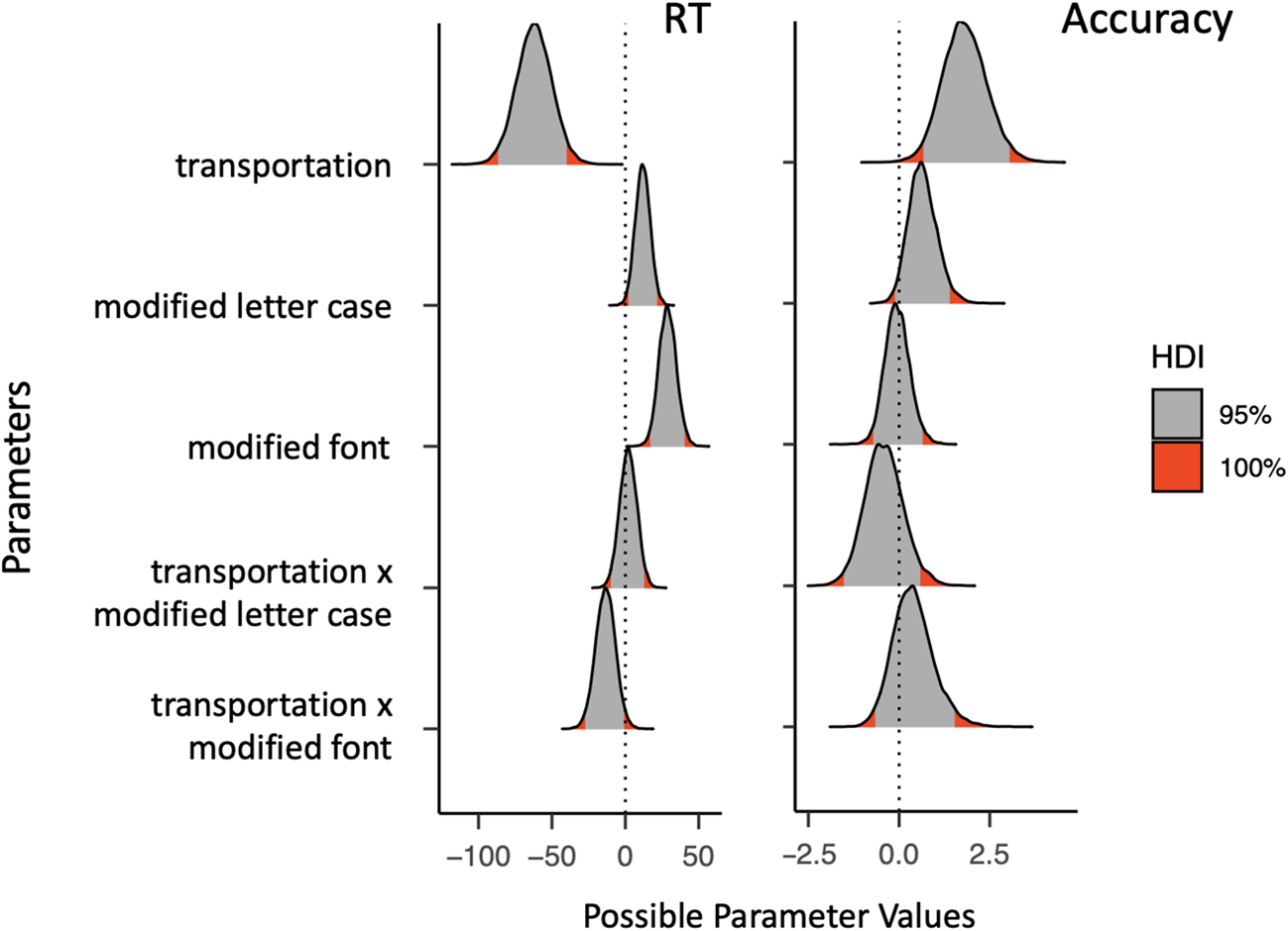
Highest density intervals with the 95% and 100% credible intervals of the posterior distributions for each of the estimates of the Bayesian linear mixed-effects models on response time (**left panel**) and accuracy (**right panel**) in Experiment 2

Participants made more errors with transportation brands than non-transportation brands (*b* = 1.81, *Estim.Error* = .61, *95% CrI* [.67, 3.05]). We found no difference between the intact logotypes and those with a modified font or letter case or interactions with the type of brand names (see Fig. 4).

For the sake of completeness, we conducted a parallel Bayesian linear mixed models analysis for the small set of 12 filler trials: six non-transportation brands embedded in transportation logos (e.g., *Ray Ban* with the graphical design of *Flixbus*) and six transportation brands embedded in non-transportation logos (e.g., OUIGO embedded in the graphical logo of PUMA). These exploratory analyses only showed slower and more error-prone responses for transportation items (response times: *b* = -61.13, *Estim.Error* = 29.33, *95% CrI *[-119.08, -2.46], accuracy: *b* = 4.67, *Estim.Error* = 1.34, *95% CrI* [2.10, 7.40]). For the filler non-transportation brands, the response times and error rates were 720 ms (4.3%), 716 ms (4.3%), and 725 ms (4.0%) for the intact brand name, the modified letter case, and the modified font versions, respectively. For the filler transportation brands, the response times and error rates were 780 ms (48.0%), 780 ms (47.3%), and 839 ms (43.7%) for the intact brand name, the modified letter case, and the modified font versions, respectively. The overall high error rates for the transportation brands should be taken with caution: three of the six filler transportation brands produced reasonably low error rates, and the high error rates were from three lesser known transportation companies in Spain (OUIGO [a French high-speed train company that runs in Spain since 2021], sixt [a rental-car company], and KLM [a Dutch airline that flies to seven cities in Spain]). In the case of OUIGO, which was embedded in the graphical design of PUMA (  ), the error rates were around 62–66% across format conditions. The more extreme case applied to KLM (the Dutch airline company), which was embedded in the Zoom graphical scheme (  ), and in which the error rates were around 76–80% across format conditions. Perhaps the dominance of certain brand logos (e.g., the transportation brand names OUIGO or KLM, when embedded in highly familiar non-transportation graphical schemes such as PUMA and Dropbox) can influence how other brands, especially less known ones, are identified when embedded within them. However, the current experiment was not designed to examine this question.

This experiment compared intact brand names embedded in their logotypes with brand names with a modified letter case or a modified font in a semantic categorization task (means of transportation or not). We found a cost for those brand names with a manipulated font or letter case relative to their intact counterparts, thus generalizing the data from the lexical decision task of Experiment 1 (i.e., is the item a brand name?) to a semantically based task (i.e., does the brand name correspond to a means of transportation?). Thus, these findings reinforce the idea that the cost measured in previous experiments using brand names, including Experiment 1, can be attributed to a general processing mechanism rather than a task-dependent artifact of the lexical decision task.

Finally, in the present semantic categorization experiment, our focus was only on the modification of the letter case or font of the original brand names while keeping the graphical design – there was only a small proportion of filler trials in which the brand name was embedded with the graphical design of a logotype of the other category (transportation vs. non-transportation brand). While beyond the scope of this paper, an interesting avenue to examine how graphical elements interact with textual information in the identification of brand names would be to examine the behavioral and electrophysiological responses to brand names when embedded in logos of the same category (e.g., IBERIA with the graphical design of Lufthansa, another transportation company) or a different category (e.g., IBERIA with the graphical design of McDonalds).

## General discussion

Most leading accounts of visual word recognition assume that perceptual elements such as font or letter case are abstracted out during lexical access (abstractionist accounts, e.g., Dehaene et al., 2005; Grainger et al., 2008; Norris, 2006). We tested whether this view is tenable for a particular class of words: brand names embedded in logotypes. Indeed, one could argue that, due to logotypes being usually presented with the same format, their representations could be more easily modulated by the perceptual elements such as font or letter case (i.e., instance-based accounts; e.g., Goldinger, 1998; Jamieson et al., 2022; Tenpenny, 1995). To disentangle the predictions of abstractionist and instance-based accounts in the identification of logotypes, we used the same set of brand names across two different tasks, one in which participants had to decide whether the brand name was real or not (i.e., an analog of the lexical decision task, Experiment 1), and one in which participants had to decide whether the brand name referred to a means of transportation (i.e., a semantic categorization task, Experiment 2). In both tasks, we found longer response times for brand names with a modified letter case and those with a modified font versus the intact brand names. Following the principles of functional overlap across tasks (Grainger & Jacobs, 1996), the similarity of the observed findings in the two tasks can be interpreted as reflecting a common process due to “visual word recognition.” We now examine the implications of these findings for theoretical accounts of visual word recognition, focusing on abstractionist, episodic, weakly abstractionist, and multiple systems accounts.

The present results revealed that identifying modified brand names in font or letter case (e.g.,  or  ) is slower than identifying intact brand names (e.g.,  ) in both lexical decision and semantic categorization tasks. These findings challenge the widespread assumption that visual word recognition is exclusively based on activating case- and font-invariant abstract letter and word units. If the memory traces of brand names were only constituted by a combination of abstract letter units – as proposed by abstractionist models (e.g., Coltheart et al., 2001; Dehaene et al., 2006; Grainger et al., 2008; Norris, 2006), one would have predicted similar response times for the intact and modified brand names in the two experiments. Nonetheless, as suggested by a Reviewer, abstractionist accounts, when applied to brand names and logotypes, might be extended to include the association between the visual aspects of words and their semantic meanings. This view, while seemingly deviating from traditional abstractionist accounts, emphasizes the significance of surface-level details in identifying brand names (e.g., letter transposition effects are stronger when logotypes are presented with their original font than with a modified font; see Perea et al., 2021, for discussion).

Overall, the present findings favor a more general approach often used to account for word identification, such as the instance theory (Goldinger, 1998; see also Ans et al., 1998; Kwantes & Mewhort, 1999; Reichle et al., 2022; Wagenmakers et al., 2004). In the context of visual word recognition research, the general idea is that words are stored and accessed through memory traces that are based on previous encounters with those words (Goldinger, 1998; Tenpenny, 1995), an idea that goes back to Semon (1923, as cited by Hintzman, 1986, 1988). Critically, the characteristics of these memory traces may depend on the use of the word and the context in which it appeared: The more frequently words are presented with different formats, the more memory traces are accumulated for that word, thus naturally explaining why high-frequency words are identified faster than low-frequency words (Goldinger, 1998) or why words that appear in many contexts are identified faster than words that appear in few contexts (Jones et al., 2012). Based on these ideas, Goldinger (1998) argues that it is possible that the memory traces of common words would be robust to changes in font and letter case, and therefore, they would act as *functionally abstract* (Goldinger, 1998; Tenpenny, 1995; for computational implementations of that idea, see Ans et al., 1998; Kwantes & Mewhort, 1999; Mikolov et al., 2013; Wagenmakers et al., 2004). Conversely, words that appear in specific contexts and formats, such as brand names, would be functionally episodic, thus being more sensitive to perceptual effects. An advantage of the principles of the instance theory is that they hold in various areas of cognitive psychology, including associative learning, human memory, spoken word recognition (see Clapp et al., 2023; Palmeri et al., 1993), and language processing (see Jamieson et al., 2022, for a review), thus providing a highly comprehensive framework.

While the present data favor instance-based over abstractionist accounts of word identification when accounting for brand name identification, we should also indicate that other accounts can capture the present findings as well, such as the weakly abstractionist and multiple system accounts. For these accounts, word recognition functions through an interplay between abstract, higher-level, and detailed, perceptual-level representations (Bowers, 2000; Marsolek, 2004; Tenpenny, 1995), possibly encoded in various perceptual subsystems (Marsolek & Burgund, 2008). In this way, these accounts acknowledge a certain level of flexibility in the visual word recognition system, where both abstract word forms and specific visual features contribute to letter and word identification. Brand names are stimuli in which perceptual information – including font or letter case – are considered relevant features, and they may be given more attention in the storage and retrieval of their internal representations. In contrast, for common words, more relevance would be given to abstract letter identities (see Bowers, 2000; Tenpenny, 1995). This explanation leaves the possibility open that both views, abstractionist and instance-based accounts, may contribute to the neural mechanisms behind visual word recognition (Bowers, 2000). The exact mechanisms behind this interplay between these two accounts are yet to be determined in future research, which may require going beyond behavioral measures (e.g., via neuroimaging studies).

Thus, the present experiments favor the view that the mental lexicon does not represent brand names as entirely abstract units. Indeed, while some specific details on logotypes may be blurry (see Blake et al., 2015), we all know that the brand name *IKEA* is usually encountered in uppercase, using blue and yellow colors and a bolded font. This observation is consistent with the idea that memory traces of stored words in the mental lexicon contain information about the circumstances in which they were encountered (Jamieson et al., 2022). In the case of words with little visual variability, such as brand names, the perceptual information has a prominent role, thus explaining why the identification of the brand name  is slower than when the brand name is presented intact (i.e.,  ). Our findings also align with empirical evidence reported with other types of words with a prevalent format, such as acronyms (e.g., *FBI* responded to faster than *fbi*; Henderson & Chard, 1976), city names (e.g., *Barcetona* is more error-prone than *Barcesona*; Perea et al., 2024), and words with initial letter capitalizations (e.g., *Mary* enjoys some processing advantage over *mary*; Jacobs et al., 2008; Labusch et al., 2022; Sulpizio & Job, 2008; Peressotti et al., 2003; Wimmer et al., 2016). Hence, our findings favor the idea that surface elements from the mental representations can be relevant in retrieving lexical information, in particular when there is a training regime in which the surface details are consistent, as occurs with brand names (e.g., Rocabado et al., 2023; see also Baciero et al., 2023, for a similar argument regarding braille words). Instead, the role played by surface details is much more limited for common words (e.g., see Perea et al., 2018, 2020, for recent evidence). All in all, these findings favor the claims made by instance theories, weakly abstractionist and multiple systems accounts of visual word recognition (see Bowers, 2000; Marsolek, 2004; Tenpenny, 1995).

In sum, the present series of experiments revealed longer identification times for modified brand names (font or letter case) embedded in logotypes than for the intact brand names in a brand identification task (Experiment 1) and a semantic categorization task (Experiment 2). These findings rule out strong abstractionist accounts of visual word recognition, for which font and letter case are “irrelevant” parameters, and, instead, favor those accounts that assume that at least under some circumstances (e.g., brand names), their memory traces in lexical memory contain relevant perceptual information that can help their identification (e.g., instance-based models, weakly abstractionist accounts, multiple systems accounts). Critically, instance-based accounts reflect a universal principle of memory functioning common to other areas of human cognition, including memory retrieval, associative learning, and spoken word recognition (see Jamieson et al., 2022, for review). The present paper has shown that the same principles can apply to written word recognition, particularly for brand names embedded in logotypes.

## Appendix 1: Pre-experiment study on the familiarity of brand names

In this pre-experiment study, ten additional participants (mean age = 26.7 years, SD = 4.6 years, four self-identified as women) were asked to rate 106 commonly known brand names according to their familiarity on a scale from 1 = “completely unfamiliar” to 5 = “completely familiar.” As a control, we also included nine unknown brand names. The average familiarity ratings per item gave us a familiarity index for each brand name. We selected the highest-scoring brand names for our study. All non-transportation brand names had a familiarity index of at least 4.7 out of 5 (mean familiarity index = 4.85). A third of the items consisted of transportation brands to fulfill the task requirements of Experiment 2. With fewer options, the transportation brands showed a familiarity index of at least 2.5 out of 5 (mean familiarity index = 3.89).

## Appendix 2: Materials used in Experiments 1 and 2

### Existing brand names (used in Experiments 1 and 2)

*Non-transportation brand names (30 items):* Adidas, Airbnb, Amazon, Carrefour, Chupa-chups, DECATHLON, DIESEL, Disney, Dominos, Duolingo, Estrella, Facebook, Google, HUAWEI, IKEA, Instagram, Intel, LEROY-MERLIN, Linkedin, Microsoft, NETFLIX, NIKE, Nintendo, Nutella, Playstation, Skype, SONY, Spotify, Vodafone, ZARA

*Transportation brand names (12 items):* AIRFRANCE, Avianca, BRITISH-AIRWAYS, Easyjet, Europcar, IBERIA, Lufthansa, Renfe, RYANAIR, TURKISH-AIRLINES, Uber, Vueling

### Non-existing brand names (42 items used in Experiment 1)

Blazz, BORIK, Cogni-zen, Commutia, Findigo, FIZO, Glynteria, Jaidi, JENDRO, Jolty, Mercabop, Molep, Moruka, Navo-celea, NEXTRONIX, NIMBUS-EQUINOXIA, Orizena, Palatious, Plix-airlines, Pluvios, Publiland, Quorazio, Quorios, RAKIA, Reparayo, Rosellea, SCHNEIF, SYLGON, TIVET, VALTORIA-QUINTUM, Vekora-airways, VELO-VORTEX, Veloza, VENOLIFE, Vixonas, Vynclara, VYNZ, Yumello, Zaluma, ZELAR, Zorvista, Zulara

## Footnotes

1 The brands were edited so that they occupied the same visual space on the screen. As a result, when the original brand name was in lowercase, the size of the letters of the corresponding uppercase condition had to be slightly reduced to occupy the same horizontal space as the original brand names and vice versa. It is important to mention that font size only plays a role in visual word recognition when it is extremely small (Chauncey et al., 2008; Morris et al., 2002).

2 The relatively high error rate for the filler trials than the experimental brands or the non-existing brands reported in Table 2 is partly due to the error-prone brand names Sixt and Zoom embedded in fake logos (  ,  , 63.3% and 24.7% of errors, respectively) and the non-existing brand name KARTZ embedded in the ROLEX logo (  , 18.7%).

3 As filler stimuli, we employed an additional set of 6 brand names belonging to a transportation company were presented within a logo of a brand that does not belong to a transportation company (e.g., the transportation brand *Flixbus* was written in the non-transportation logo of *Dropbox*) and six brand names not belonging to a means of transportation were presented within a logo of a brand that does belong to a means of transportation (e.g., the non-transportation brand *Ray Ban* was written in the transportation logo of *Flixbus*; see Fig. 3 for examples). Similar to the experimental stimuli, the filler brand names were presented in the three versions (original, modified letter case, modified font).
